# DNA Recovery Using Different Extraction Kits and Cotton Swabs in Forensic DNA Analysis

**DOI:** 10.3390/genes17040457

**Published:** 2026-04-14

**Authors:** Ghassan Ali Salih, Martina Nilsson, Marie Allen

**Affiliations:** 1 Department of Immunology, Genetics and Pathology, Uppsala University, Box 815, S-751 08 Uppsala, Sweden; ghassan.salih@igp.uu.se (G.A.S.); martina.nilsson@polisen.se (M.N.); 2 Department of Biology, College of Science, University of Baghdad, Baghdad 10071, Iraq; 3 Swedish Police Authority, Stockholm Region, Forensic Unit, S-106 75 Stockholm, Sweden

**Keywords:** forensic trace DNA, DNA extraction, cotton swab sampling, forensic DNA analysis, STRs, mtDNA

## Abstract

**Background:** It is essential to recover as much DNA as possible from evidence samples to ensure optimal DNA analysis in forensic casework. However, both DNA collection and purification procedures cause a substantial loss of genetic material. Thus, a large loss of DNA through the pre-PCR procedures, including swabbing and extraction, may significantly affect downstream analysis results. In this study, different cotton swabs and extraction kits used for forensic samples were compared separately. **Methods:** The recovery of cell-free DNA (control DNA) and cell-bound DNA (blood and saliva) was evaluated using five different extraction kits: Chelex^®^ 100 Resin, Wizard^®^ Genomic DNA Purification Kit, QIAamp^®^ DNA Micro Kit, QIAamp^®^ DNA Investigator Kit and DNeasy^®^ Blood & Tissue Kit. The DNA recovery efficiency of the different extraction kits was assessed using real-time quantitative PCR targeting nuclear and mitochondrial DNA targets. In addition, nine cotton swabs from four manufacturers (Selefa^®^, Puritan^®^, Texwipe^®^, and Heinz Herenz) with different production lots were evaluated for DNA quantity and quality using real-time PCR and short tandem repeat (STR) analysis. **Results:** Overall, large differences in DNA recovery were observed between the different extraction kits. The QIAInvestigator kit demonstrated the highest recovery at low DNA amounts, which is particularly beneficial for minute forensic samples. The swab comparison revealed variations not only in DNA recovery between swab manufacturers but also between lots of the same swab brand, and the DNA quantity was not clearly correlated with downstream DNA profile quality. **Conclusions:** Our findings emphasise the importance of considering the choice of extraction kit, swab brand and batch-to-batch variation in forensic laboratory procedures, as they may influence DNA recoveries and affect the success rate in forensic casework.

## 1. Introduction

Analysis of forensic DNA evidence has become an important part of criminal investigations, and routine analysis relies on generating a DNA profile from multiple short tandem repeat (STR) markers. Several commercial STR typing kits recommend DNA input as low as 500 pg, but lower input may yield results [1,2,3]. However, when cellular material is scarce or degraded, and the quantity is much lower than the recommended input threshold, the sample is defined as Trace DNA [4]. As alternative approaches, additional PCR cycles or mini STR markers may improve profiling results for Trace DNA samples [5,6,7,8]. Moreover, when the amount of nuclear DNA (nDNA) is too scarce to produce STR profiles, mitochondrial DNA (mtDNA) analysis may be successful because of the high abundance of mtDNA copies present in each cell [9]. Forensic samples may consist of different tissue types, including body fluids such as blood, saliva, and semen, which are often found at crime scenes and are routinely used for DNA analysis. In addition, so-called touch DNA stemming from skin cells is commonly sampled. Approximately 400,000 skin cells are shed by an individual daily, containing DNA that could be transferred when an object is handled or touched [10]. 

Trace DNA often consists of skin cells transferred to touched items (e.g., tools, clothing, firearms, paper, glass, or wood) and has proven useful for DNA analysis in crime scene investigations [11,12,13]. It has, for example, been demonstrated that DNA profiles can be generated from epithelial cells found on touched items, such as a gun, cartridge, paper, glass rim, doorbell and a cigarette lighter [10,11,14,15]. Trace DNA analysis has therefore become an important tool for obtaining evidence in severe crimes such as sexual assaults, murders, and robberies. Apart from cell-bound DNA (cbDNA) within the nucleus, touch DNA samples also contain a considerable amount of cell-free DNA (cfDNA), resulting from apoptosis, DNA degradation, and environmental exposure [16]. Several factors influence the retrieval and persistence of DNA from touched evidence materials, which typically contain low amounts of DNA. DNA recovery is, for example, affected by the duration between contact and sample collection, pressure, friction, donor perspiration, donor shedding status, and hand-washing routines [17,18,19]. The surface type of the handled items has also been shown to play a crucial role in DNA recovery. Several studies have investigated the effects of porous and non-porous materials on DNA recovery, reporting significant differences [20,21].

While many studies have focused on improving PCR amplification efficiency and typing kit performance, the initial sampling and extraction procedures may still rely on procedures that yield poor DNA recovery. Forensic laboratories use various extraction methods, including organic solvents, silica particles, chelating resins, salting-out procedures, or magnetic beads [22]. Chelating resins, such as Chelex, use ion-exchange technology to bind polar cellular components and metal ions, whereas other methods, such as the QIAamp^®^ DNA Kits, use silica-based membranes and serial binding for DNA purification. Additionally, the salting-out method used in the Wizard^®^ Genomic DNA purification kit precipitates cellular proteins at high salt concentrations, leaving the DNA in the supernatant. It has been demonstrated that when using Chelex, 42–76% of the DNA is lost, whereas with an organic extraction method, 45–75% is lost [23].

In addition to DNA loss during extraction, previous studies have shown that forensic DNA analysis is influenced by the DNA collection device [14,24]. Cotton or nylon swabs are commonly used to collect DNA from a variety of biological materials, and DNA recovery varies with the swab material used [24,25,26]. Cotton swabs are the most widely used sampling tool, but several drawbacks have been noted that may compromise the quality of forensic DNA analysis. The polymerisation of cellulose makes cotton rich in hydroxyl (-OH) groups, which can form hydrogen bonds with DNA, and prevent its release into the extraction solution. Additionally, cotton may shed fibres into the extraction solution, acting as an inhibitor during real-time quantitative PCR (qPCR) and downstream DNA profiling [27]. Moreover, the presence of hydroxyl (-OH) groups in cotton swabs may vary among manufacturers due to differences in cotton sourcing, processing techniques, and chemical treatments, all of which can influence the interaction between the swab and the biological sample. These variations may occur not only between manufacturers, but also across different production years or batch (lot) numbers from the same producer, potentially affecting the DNA retention and sample release efficiency of the swabs. While several studies have evaluated the efficiency of cotton swabs from different manufacturers, limited research has examined variability in swab performance across production batches from the same manufacturer.

This study was designed around two main objectives, each of which was addressed independently. The first objective was to evaluate and compare the efficiency of various DNA extraction kits, and the second was to investigate the impact of different cotton swabs and lot-to-lot variability on DNA recovery. These two factors, the extraction method and DNA collection device, are inherently interconnected and will significantly influence the final yield and quality of DNA for downstream applications. The extraction procedure comparison involved five different extraction kits: Chelex^®^ 100 Resin (Chelex), Wizard^®^ Genomic DNA Purification Kit (Wizard), QIAamp^®^ DNA Micro Kit (QIAMicro), QIAamp^®^ DNA Investigator Kit (QIAInvestigator) and DNeasy^®^ Blood & Tissue Kit (QIADNeasy). Their efficiency was assessed using real-time PCR to quantify nDNA and mtDNA extracted from simulated forensic-type samples, including cbDNA from blood and saliva, and cfDNA in the form of an extract at three concentrations. In addition, nine cotton swabs were evaluated to determine the effects on DNA recovery and downstream analysis. Specifically, different swab production lots from four manufacturers were tested: Selefa^®^ (three lots), Puritan^®^ (three lots), Texwipe^®^ (two lots), and Heinz Herenz (one lot). Swab performance and sample release were evaluated using both real-time qPCR and STR typing.

## 2. Materials and Methods

### 2.1. Extraction Kit Comparisons

#### 2.1.1. DNA Samples

The TaqMan^®^ Control Genomic DNA sample (Applied Biosystems, Waltham, MA, USA) was used in experiments simulating cfDNA. A total of 10 µL cfDNA was applied to the non-adhesive side of brown packing tape in a circular area with a diameter of 25 mm. The cfDNA was diluted to 6 ng/µL, 0.6 ng/µL, and 0.06 ng/µL. The three different concentrations resulted in the deposition of 60 ng, 6 ng, and 0.6 ng cfDNA, corresponding to 20,000, 2000, and 200 nDNA copies, respectively. Moreover, using an average estimation of 1000 mtDNA copies per cell, a total of 20,000,000, 2,000,000, and 200,000 mtDNA copies were deposited. The mtDNA copy number for this sample was estimated in experiments by Andréasson et al. (2002) [28].

To evaluate forensic materials with cbDNA, blood and saliva samples were collected from a donor with informed consent. A total of 10 µL of blood or saliva was applied to the non-adhesive side of brown packing tape in a circular area with a diameter of 25 mm. As the blood and saliva samples have unknown DNA concentrations, the actual recovery of cbDNA could not be estimated. However, a side-by-side comparison of the extraction kit efficiency could still be performed. Samples with cfDNA, blood and saliva were spread across the entire marked area of clean brown packing tape using a pipette tip, and left to dry (approximately 1 h). All stains were recovered with cotton swabs moistened with 1% SDS (Selefa, OneMed, Helsinki, Finland) using circular movements for 10 s, maintaining constant pressure. The cotton tips were placed in Eppendorf tubes for subsequent extraction.

#### 2.1.2. DNA Extraction

Five commonly used extraction kits were utilised for the extraction: Chelex^®^ 100 Resin (Bio-Rad, Hercules, CA, USA), Wizard^®^ Genomic DNA Purification Kit (Promega, Madison, WI, USA), QIAamp^®^ DNA Micro Kit (Qiagen, Hilden, Germany), QIAamp^®^ DNA Investigator Kit (Qiagen) and DNeasy^®^ Blood & Tissue Kit (Qiagen). Their efficiency was evaluated for both cfDNA and cbDNA in blood and saliva. All extractions were carried out according to the manufacturer’s protocol. For the QIAMicro, QIAInvestigator, and QIADNeasy kits, two elution steps of 50 µL each were applied (total 100 µL), whereas Chelex had a total extraction volume of 200 µL. Negative extraction controls were included in all extractions to monitor possible contamination.

#### 2.1.3. DNA Quantification

The extracted DNA was analysed by real-time PCR using an assay that quantifies both nDNA and mtDNA. A total of 10 µL of DNA extract was used with the TaqMan^®^ Universal PCR Master Mix (Applied Biosystems), according to the manufacturer’s instructions. A 79 bp nuclear DNA fragment in the human retinoblastoma 1 gene, and a 142 bp human-specific mtDNA target were used in PCR amplification for 40 PCR cycles [28]. Negative controls (no DNA) were included in all set-ups to monitor contamination. DNA was quantified from four to five biological replicates and two technical replicates for each variable using the Applied Biosystems StepOnePlus™ Real-Time PCR System. Copy number calculations were performed using the StepOne Software v2.2 (Applied Biosystems). Strict contamination prevention routines were applied throughout all pre-PCR stages, using fully disposable protective gear, disposables [29] and appropriate cleaning routines [30].

As the final extraction volumes differ between kits (100 and 200 µL), all yield calculations were adjusted to account for the extraction volume and to represent the DNA content in the whole extract. The DNA recovery data were expressed relative to the best-performing kit (set to 100%) for both cfDNA and cbDNA to account for the unknown input copy numbers in cbDNA. For cfDNA, the DNA loss for each extraction method was calculated as the ratio of the number of copies retrieved to the copies added, since the exact input numbers were known. Moreover, in one experiment, cfDNA was added directly to the extraction tube for the QIADNeasy kit, allowing the estimation of DNA loss during the extraction procedure only, without swabbing. Data analysis was performed using Microsoft^®^ Excel (version 2021). The 1.5 × interquartile rule was applied to the four or five biological replicates for each condition (cbDNA or cfDNA and five evaluated kits) to identify outliers. A summary of recovered nDNA and mtDNA for cfDNA and cbDNA relative to the best- performing kit is presented in  Supplementary S1, Table S1. Data with and without the removal of outliers are presented in  Supplementary S1, Tables S2 and S3. Statistical differences in extraction efficiency were assessed using the Kruskal–Wallis test, followed by pairwise comparisons with Dunn’s test and Holm’s correction for multiple testing. All *p*-values are reported in  Supplementary S1, Table S4. Visualisations, including bar plots and statistical annotations, were generated in RStudio^®^ software (version 2023.06.0+421) using the ggplot2, FSA, tidyr, RColorBrewer, forcats, and dplyr packages.

### 2.2. Swab Comparisons

#### 2.2.1. DNA Samples

The sampling efficiency of various cotton swab brands was evaluated by measuring the quantity and quality of recovered DNA (Table 1). Whole blood (20 µL) from one donor was pipetted onto the non-adhesive side of brown packing tape that had been cleaned with 4.5% bleach. The stain was air-dried for 2 h at room temperature. Each swab was moistened with two drops of 0.9% NaCl, and a standard swabbing procedure was applied uniformly across all samples to ensure consistency. Four biological replicates per swab type were prepared, and negative extraction controls were included to monitor potential contamination. The swab evaluation was performed independently of the extraction kit comparison, using a single extraction method for all swabs to isolate the swab-specific effects. DNA extraction was performed using 5% Chelex 100 Resin (Bio-Rad) in a total extraction volume of 1 mL. Each extraction solution also contained 10 µL of Proteinase K (10 mg/mL; Qiagen) and 20 µL of Tween 20 (10%; Roche, Basel, Switzerland) [31].

#### 2.2.2. DNA Quantification

Each biological replicate for each swab type was analysed in triplicate using the commercial Quantifiler™ Trio DNA Quantification kit (Applied Biosystems) for nDNA quantification, following the manufacturer’s instructions. The mean of the technical replicates, the mean of the biological replicates, and total DNA recovery were calculated (Supplementary S2, Table S5). Differences in DNA recovery among swabs were evaluated using the Kruskal–Wallis test, followed by pairwise comparisons using Dunn’s test with Holm adjustment for multiple comparisons (Supplementary S2, Table S6). Additionally, bar plots of total DNA recovery with *p*-values and lollipop plots of RFU intensities for various STR markers were generated using RStudio^®^ software (version 2023.06.0+421), employing the following packages: ggplot2, FSA, rcompanion, tidyverse, forcats, dplyr, stringr, and patchwork.

#### 2.2.3. STR Analysis

The biological replicate with the DNA quantity closest to the median DNA recovery was selected for STR analysis to evaluate DNA quality (n = 9). STR profiling was performed using the VeriFiler™ Plus Kit (Applied Biosystems), with a 250 pg DNA input for PCR and a 29-cycle amplification protocol on a Veriti^®^ Thermal Cycler (Applied Biosystems). One microliter of each PCR was subjected to fragment size analysis on a SeqStudio™ Genetic Analyzer (Applied Biosystems), using the GeneScan™ 600 LIZ™ Size Standard (Applied Biosystems). STR profiles were analysed using GeneMapper™ Software v6 (Applied Biosystems), applying a 50 RFU threshold (Supplementary S2, Table S7). The STR markers in the VeriFiler Plus kit were categorised into six groups based on amplicon length: 100–125 bp, 150–200 bp, 200–250 bp, 250–300 bp, 300–350 bp, and >350 bp (Supplementary S2, Table S8). To statistically assess DNA quality, the relationship between RFU intensities of markers and marker size groups was evaluated using Pearson’s correlation analysis in RStudio (version 2023.06.0+421) using the following packages: dplyr, ggplot2, ggpubr, RColorBrewer, and forcats.

## 3. Results

### 3.1. Extraction Kit Comparisons

**Figure 1:**
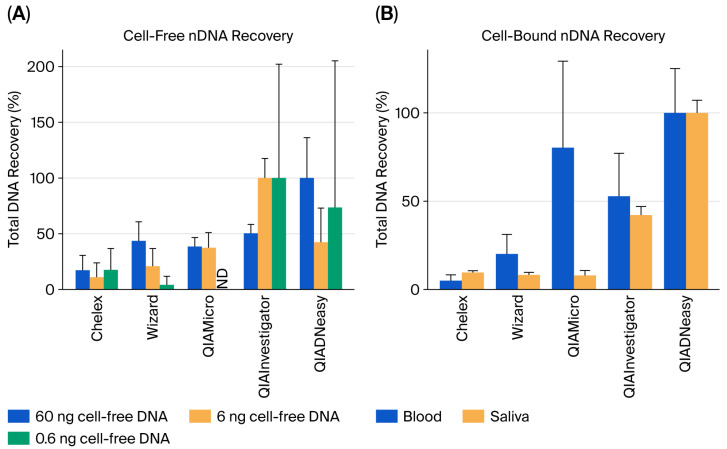
Recovery of nDNA using different extraction kits. (**A**) Cell-free nDNA in three different deposition amounts is shown as percent recovery (in relation to the best-performing kit for each deposited DNA amount). (**B**) Cell-bound nDNA in blood and saliva is shown as percent recovery (in relation to the best-performing kit for the two sample types). Error bars represent normalised standard deviation (SD %), calculated as SD % = (mean % × CV %)/100, for comparison of variation. ND—not determined. The lowest deposition amount, 0.06 ng DNA, was not analysed for the QIAMicro kit.

In this study, the recovery of cfDNA in a control sample and cbDNA in blood and saliva (with unknown DNA concentrations) was compared using five different extraction kits. Three different amounts of cfDNA were used: 60 ng, 6 ng, and 0.6 ng. The lowest amount of deposited DNA (0.6 ng) corresponds to approximately 100 cells (200 nDNA copies), a range commonly observed in entire extracts from trace DNA samples. For cell-free nDNA, recovery ranged from approximately 4% to 100% relative to the best-performing kit. The highest DNA yields were obtained using the QIADNeasy kit (60 ng deposition) and QIAInvestigator kit (6 and 0.6 ng). The Chelex, Wizard, and QIAMicro extraction methods demonstrated lower recovery rates, ranging from 11 to 17% (Chelex), 4 to 43% (Wizard), and 37–38% (QIAMicro). Chelex extraction showed the lowest recovery for the highest deposition amount, while the Wizard kit showed the lowest recovery for the lowest deposition amount. Noteably, the QIAInvestigator kit demonstrated the best recovery for the lowest DNA deposition amount, which is beneficial for forensic analysis (Figure 1A;  Supplementary S1, Tables S1 and S2). For cell-bound nDNA, the highest nDNA yields were obtained using the QIADNeasy kit for both the blood and saliva depositions. When comparing the remaining kits to the QIADNeasy kit, the recovery ranged from 5 to 80%, whereas the maximum recovery rates from Chelex and the Wizard kit were ≤20%. Additionally, the QIAMicro showed a significant difference in recovery between blood and saliva (Figure 1B;  Supplementary S1, Tables S1 and S2).

**Figure 2:**
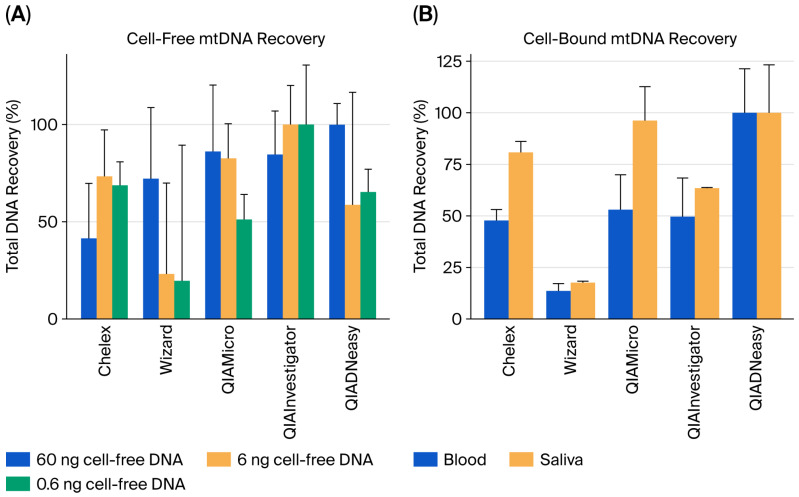
Recovery of mtDNA using different extraction kits. (**A**) Cell-free mtDNA in three different deposition amounts is shown as percent recovery (in relation to the best-performing method for each deposited DNA amount). (**B**) Cell-bound mtDNA in blood and saliva is shown as percent recovery (in relation to the best-performing method for the two sample types). Error bars represent normalised standard deviation (SD %), calculated as SD % = (mean % × CV %)/100, for comparison of variation.

Similarly to cell-free nDNA, the highest DNA yields were obtained for cell-free mtDNA using the QIADNeasy kit (60 ng deposition) and QIAInvestigator kit (6 and 0.6 ng). When comparing recovery rates against the two best-performing kits, recovery ranged from 20 to 86%. Furthermore, the QIAMirco and QIAInvestigator kits demonstrated the second-best performance at 60 ng deposition. For the lowest DNA amount (0.6 ng), the second-best recovery was observed using Chelex and QIADNeasy (Figure 2A;  Supplementary S1, Tables S1 and S3). Overall, the best recovery of the lowest DNA amount (0.6 ng) was demonstrated using the QIAInvestigator, Chelex and QIADNeasy kit. For the cbDNA in blood and saliva depositions, the highest mtDNA yields were obtained using the QIADNeasy kit. When the other kits were compared to QIADNeasy, mtDNA recoveries ranged from 14 to 96%. For cbDNA, the QIAInvestigator, QIADNeasy, QIAMicro, and Chelex extractions demonstrated high recoveries ranging from 48 to 100%, whereas the Wizard kit resulted in less than 20% recovery (Figure 2B;  Supplementary S1, Tables S1 and S3). For cell-free mtDNA, the best recovery was observed with the QIAInvestigator and QIADNeasy, while the lowest was observed with Wizard (6 and 0.6 ng).

### 3.2. DNA Loss

It is well known that both swabbing and the subsequent extraction process result in a significant loss of DNA [27,32]. Samples with a known starting DNA quantity showed mean copy-number recoveries of nDNA ranging from 1.0 to 27.3% for the lowest deposited cfDNA amount. Similarly, when quantifying mtDNA, the mean recovery of cfDNA ranged between 6 and 28.1% for the lowest deposited amount (Supplementary S1, Tables S2 and S3). Thus, the total loss during extraction and swabbing exceeds 70% of the added DNA. This significant loss of DNA will impact the analysis of forensic materials, which often contain low amounts of DNA. To separately examine the loss of DNA during extraction, we also compared recovery relative to the DNA input rather than to the best-performing kit. The recovery of known input amounts of mtDNA (in copies and ng) was used as the “maximum extraction recovery” yield. The recovery was obtained by adding DNA directly into the extraction reaction (without swabbing) using the QIADNeasy kit. The maximum recovery of mtDNA was 42.3, 35.6, and 34.4% for 60, 6, and 0.6 ng of DNA added, respectively. This shows that up to 66% of the DNA is lost during the extraction procedure alone (row *f* in  Supplementary S1, Table S3). The swabbing process is also expected to cause additional DNA loss. In this study, swabbed and extracted mtDNA yielded a total mean recovery of 23.0, 10.1, and 18.4% (60, 6, and 0.6 ng of DNA added, respectively) using the QIADNeasy kit (Supplementary S1, Table S3). This demonstrates that the swabbing process alone accounted for additional losses of 19.3%, 25.5%, and 16%. 

### 3.3. Swab Comparisons

**Figure 3:**
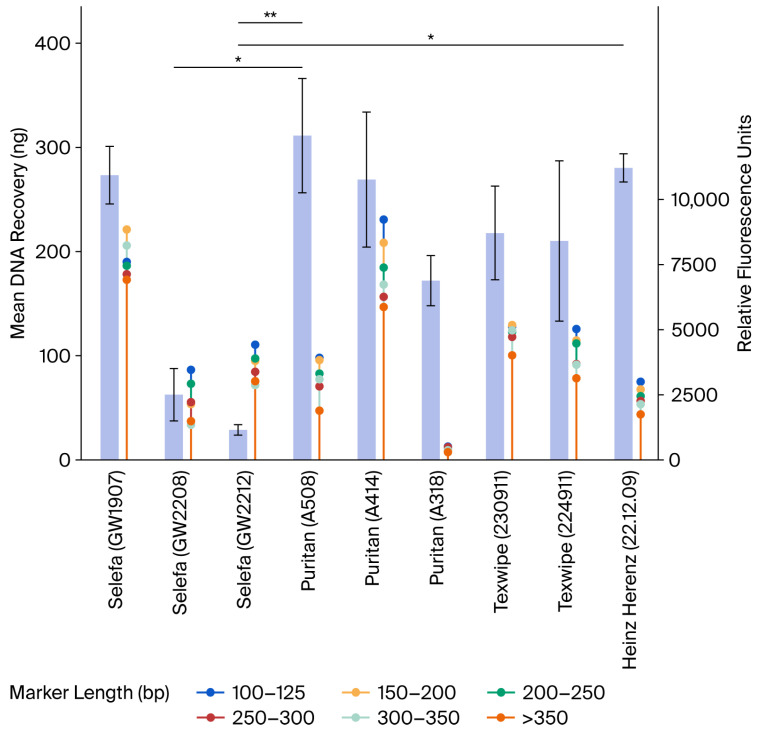
Bar plot (left *y*-axis) with the total DNA recovery from nine cotton swabs. Lollipop plot (right *y*-axis) with the RFU values for STR markers grouped after the allele lengths for the nine tested cotton swabs. * *p* < 0.05, ** *p* < 0.01.

To further investigate DNA loss during swabbing, nine cotton swabs with different lot numbers from four manufacturers were examined for DNA quantity and quality in 20 µL whole blood (Table 1). A considerable variation in total DNA recovery was observed among different swabs, as well as between different lot numbers of the same swab type (Figure 3). The amount of DNA input from 20 µL of whole blood is unknown, and the total recovery ranged between approximately 30 and 300 ng. The highest recovery rates were observed with Selefa^®^ (GW1907), Puritan^®^ (A508 and A414), and Heinz Herenz, with total DNA yields ranging from 270 to 311 ng. The remaining swabs demonstrated recoveries, ranging from 28.7 to 218 ng. Notably, the three Selefa^®^ lots demonstrated varying performance. The GW1907 lot outperformed the other two, GW2208 and GW2212, with approximately 5-fold and 10-fold higher DNA yields, respectively (Supplementary S2, Table S5). Similarly, while two Puritan^®^ lots (A508 and A414) performed comparably, the third lot (A318) yielded substantially less DNA. Both lots of Texwipe^®^ swabs yielded nearly equivalent DNA amounts, with total recoveries of 218 and 210 ng. Moreover, the comparison of all nine swabs revealed significant differences, with *p*-values < 0.05 between Puritan^®^ A508 and Selefa^®^ GW2208, Puritan^®^ A508 and Selefa^®^ GW2212, and Hienz Herenz and Selefa^®^ GW2212 (Supplementary S2, Table S6).

**Figure 4:**
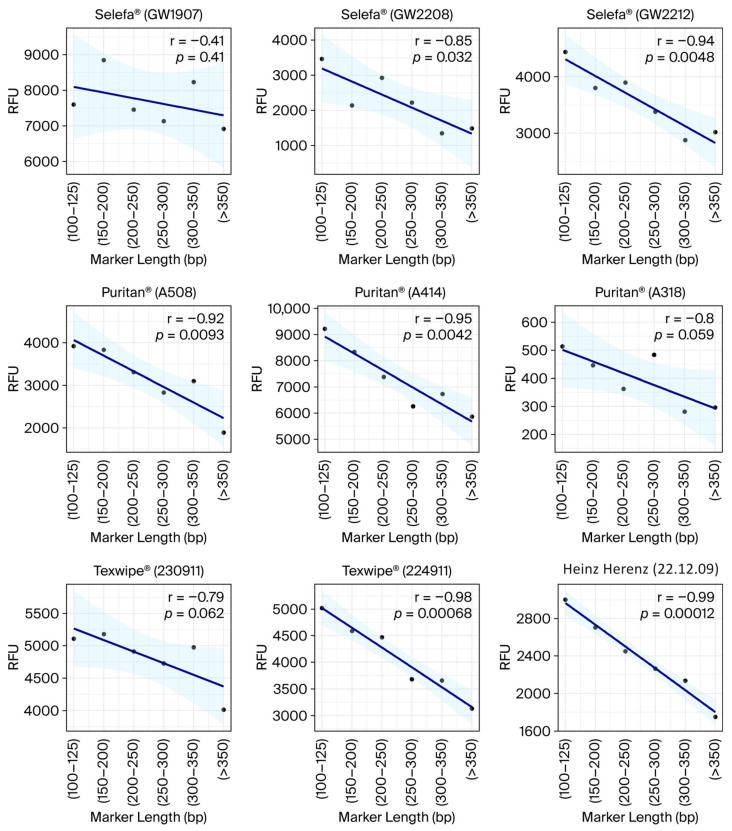
Pearson’s correlation (r) between RFU values and grouped STR marker size ranges obtained using the VeriFiler™ Plus kit across the nine different swabs.

An STR analysis (n = 9) was conducted to evaluate the quality of DNA collected with different swabs, on the biological replicate closest to the median DNA yield for each swab type. A comparison between RFU values and amplicon sizes was performed to assess whether reduced amplification of larger-size loci occurred, which would indicate size-dependent inhibition. In general, there was a negative correlation between RFU values and marker sizes across the tested swabs, with r values ranging from −0.41 to −0.99, indicating a size-dependent reduction in signal (Figure 4; Supplementary S2, Table S9). The RFUs ranged from approximately 500 to 9000 for the shortest markers and 300 to 7000 for the longest markers, with large variation between swabs. The strongest negative correlations were observed for Puritan (AA414), Texwipe (224911), and Heinz Herenz, with r values of −0.95, −0.98, and −0.99, respectively. The Selefa (GW1907) and Puritan (A414) swabs yielded both very high DNA quantities and RFUs, while the Selefa (GW2808) and Selefa (GW2212) swabs yielded notably lower DNA quantities with higher RFUs relative to the concentration (Figure 3 and Figure 4; Supplementary S2, Tables S5 and S7–S9). Moreover, Puritan (A508), Puritan (A318), and Heinz Herenz yielded high DNA quantities and lower RFUs. Interestingly, these swabs yield low RFU signals relative to the DNA recovery, indicating that high DNA quantity is not always correlated with downstream amplification success. A summary of observed degradation, including size-dependent signal loss among the tested swabs, is presented in Supplementary S2, Table S9. The overall findings reveal that DNA recovery varies not only across swab brands but also between different lots of the same brand.

## 4. Discussion

DNA recovery and loss remain major challenges in forensic casework, often resulting from sample collection and DNA extraction. This study first evaluated five DNA extraction kits and assessed the DNA recovery. The QIADNeasy Kit yielded the highest nDNA and mtDNA recovery for blood, saliva and cfDNA (60 ng). However, at 0.6 ng cfDNA, the QIAInvestigator kit demonstrated the highest recovery for both nDNA and mtDNA. Across different kits, 65–99% of cell-free nDNA and 72–96% of cell-free mtDNA were lost during the full workflow, including swabbing (with the same swab type). These findings are consistent with previous DNA extraction studies reporting 20–84% loss during combined sampling and extraction procedures [23,33,34]. To further differentiate loss during sampling and extraction, we added cfDNA directly to the QIADNeasy extraction tube, and found that 58–66% of the deposited material was lost during extraction, whereas swabbing alone accounted for an estimated 16–26% loss. Although this experiment involved only one kit, similar losses by swabbing are likely with the other Qiagen kits, as the process is equivalent. Thus, most of the differences in this study appear to be attributable to the extraction procedures.

Forensic samples vary widely in composition, quality, and quantity, and both cbDNA and cfDNA may be present in unknown proportions. Therefore, simulated stains (blood and saliva) and cell-free DNA were used to evaluate recovery, representing both types of DNA. It should also be noted that none of the extraction kits evaluated in this study was developed for cfDNA, unlike, for example, the QIAamp Circulating Nucleic Acid Kit. Nonetheless, comparing these kits still provides guidance for future decisions on extraction kit choices. In addition, the sampled surface was standardised using adhesive tape to ensure comparability across evaluated extraction kits and swabs. As substrate properties can influence DNA retention and recovery efficiency [35,36], the findings should be interpreted in light of this study design. 

A second limitation of this study is that downstream STR analysis was not performed. The data presented are based on real-time qPCR quantification of a 79 bp fragment of nDNA and a 142 bp fragment of mtDNA. Although high DNA quantities obtained in a real-time quantification assay do not necessarily ensure successful downstream STR or mtDNA analysis, as previously demonstrated by the lack of a clear correlation between PCR product size and starting copy numbers [37], the extraction kit comparison in this study was conducted under controlled experimental conditions. A highly purified reference DNA (TaqMan^®^ Control Genomic DNA) was used for the cfDNA experiments, and DNA was efficiently extracted from high volumes of blood and saliva deposits for the cbDNA experiments. The samples were therefore expected to be free of PCR inhibitors and DNA fragmentation. However, potential differences in downstream success between kits were not fully assessed and present a limitation in this study, but may be addressed in future investigations.

Swab-related variability was also assessed by evaluating nine cotton swabs from four manufacturers. Real-time qPCR was used for DNA quantification, and STR profiling was performed to assess profiling success, degradation and PCR inhibition. DNA recovery varied across swabs from different manufacturers, and between different lots of the same product, likely reflecting differences in cotton, processing conditions, or the presence of inhibitory compounds introduced during manufacturing. The physical characteristics of the cotton, such as weave type, thread count, and thread orientation, may influence DNA quantity and quality [38]. Previous studies have compared different swab types [26,39], but to our knowledge, no study has examined batch performance within the same swab brand.

Among the tested swabs, Selefa^®^ (GW1907) yielded the highest DNA recovery, whereas other lots of the same brand showed lower recoveries. Notably, this lot had passed its expiration date at the time of testing, yet it demonstrated the highest DNA recovery and good STR results. While the potential influence of storage time cannot be entirely excluded, the findings suggest that batch-related variability rather than expiration status was the primary contributor to the observed differences. Moreover, the relationship between RFUs and STR marker sizes for Selefa^®^ (GW2208) and Selefa^®^ (GW2212) showed strong negative correlations, indicating potential lot-specific inhibition issues. The Puritan^®^ lots did not differ significantly in yield, yet RFU intensities varied and did not correspond to the recovered DNA quantities, reinforcing that genotyping success does not always align with DNA quantity. Also, Heinz Herenz had high DNA recovery but showed strong negative correlations between RFU values and STR marker size ranges, and overall lower RFUs than Selefa^®^ swabs (lot GW1907). Furthermore, swabs from the same manufacturer may produce consistent quantities across lots, but differ in STR performance. For example, two Texwipe^®^ lots yielded similar DNA quantities but showed different degradation patterns in STR profiling. Thus, the qPCR and STR results show that high DNA recovery does not necessarily translate to high genotyping success. Previous research on inhibitors has demonstrated reduced amplification of longer alleles and a degradation pattern while minimally affecting qPCR Ct values [40,41]. Such discrepancies can be misleading when determining suitability for downstream applications such as STR profiling.

## 5. Conclusions

This study quantified DNA loss associated with sampling and extraction procedures, but did not aim to identify the optimal swab–extraction combination. Both the choice of extraction kit and the swab brand, including production lot, substantially influenced DNA recovery, especially for low-copy-number samples. Although not all commercially available kits and swabs were evaluated, the findings highlight that methodological decisions at both the sampling and extraction stages affect DNA yield and the interpretation of DNA profiling results. Significant DNA losses were observed across swab brands and lots of the same brand, and high DNA amounts did not always correlate with high STR success. The observed batch-related variability underscores the importance of production quality control and transparency from manufacturers, especially following changes to production procedures. Based on these findings, our laboratory will adopt the QIAamp^®^ DNA Investigator kit for routine casework, as it demonstrated the best recovery under low-template samples. We will also implement systematic lot-to-lot assessments of swab performance and DNA release to enhance consistency and reliability in forensic DNA analysis.

## Figures and Tables

**Table 1:** List of the swabs evaluated and their lot numbers, manufacturer reference number, expiry date, and manufacturer.

Swab	Lot Number	Manufacturer Reference	Expiry Date	Manufacturer
Selefa^®^	GW1907	120788	June 2022	OneMed Group (Stockholm, Sweden)
Selefa^®^	GW2208	120788	July 2025	OneMed Group
Selefa^®^	GW2212	120788	November 2025	OneMed Group
Puritan^®^	A508	25-806 1WC FDNA	June 2028	Puritan Medical Products (Guilford & Maine, MN, USA)
Puritan^®^	A414	25-806 2WC	April 2028	Puritan Medical Products
Puritan^®^	A318	25-806 1WC FDNA	February 2028	Puritan Medical Products
Texwipe^®^	224911	STX705W	December 2025	Texwipe^®^ An ITW Company (Kernersville, NC, USA)
Texwipe^®^	230911	STX705P	March 2026	Texwipe^®^ An ITW Company
Heinz Herenz	22.12.09	1030419	September 2027	Heinz Herenz, Medizinalbedarf GmbH (Hamburg, Germany)

## Data Availability

Data are contained within the article and Supplementary Materials.

## Supplementary Materials

The following supporting information can be downloaded at https://www.mdpi.com/article/10.3390/genes17040457/s1: Table S1. Summary of mean nDNA and mtDNA recovery in percent using different extraction kits in relation to the best-performing kit; Table S2: nDNA recovery; Table S3: mtDNA recovery; Table S4: Kruskal-Wallis tests of DNA recoveries using different extraction kits, followed by pairwise comparisons using Dunn’s test with Holm adjustment for multiple comparisons (values have been correlated to the final extraction volume); Table S5: Quantification results for the three technical replicates of each of the four biological replicate, median values, mean values, standard errors, standard deviations for the four biological replicates of each swab, and samples subjected to STR analysis; Table S6: Kruskal-Wallis tests of DNA recoveries from different swabs, followed by pairwise comparisons using Dunn’s test with Holm adjustment for multiple comparisons; Table S7: RFUs for generated STR profiles from different swabs. All swabs were tested on a blood sample from same donor; Table S8: Allele sizes in bp of STRs in the Verifiler Plus kit according to allele length groups, with mean values of each allele-length group for the nine tested swabs; Table S9: Observation of ski-slope patterns and size-dependent signal loss for the tested swabs, including Pearson correlation coefficients (r) and associated *p*-values.
